# Can solidarity in global health curb the next outbreak? A commentary on mpox

**DOI:** 10.1136/bmjgh-2024-018116

**Published:** 2025-03-07

**Authors:** Natalie Tegama, Julian Natukunda, Imogen Alexandra Fiona Brown, Caesar Alimisnya Atuire

**Affiliations:** 1 Centre for Tropical Medicine and Global Health, Nuffield Department of Medicine, University of Oxford, Oxford, UK

**Keywords:** Global Health, Infections, diseases, disorders, injuries

**SUMMARY BOX**

- Solidarity rhetoric is a constant feature in global health; however, solidarity is an ill-defined concept that is differently understood and practiced across contexts.
- We bring some conceptual clarity by introducing *deep* and *responsive* solidarity. We trouble responsive solidarity in relation to the current mpox outbreak and show why deep solidarity would be more instrumental in helping us curb outbreaks.
- Developing better conceptualisations of solidarity is imperative for global health.

Solidarity rhetoric has become a regular feature in global health, particularly in the context of global health emergencies. It is, however, a nebulous concept, differently defined, understood and practiced around the world, from familial contexts to the state and international levels. In the most recently declared Public Health Emergency of International Concern (PHEIC)^1^ mpox, we have seen calls for solidarity towards the epicentre, the Democratic Republic of Congo (DRC) and the broader African region. These calls for solidarity have been largely driven by global health scholars and the Africa Centre for Disease Control.^2^,^4^ The tardy and insufficient response^5^ to these calls contribute to a poignant example of gaps in both our understanding and our practice of solidarity within the international community. It is often the case that the underlying conceptualisations of solidarity between Global South stakeholders are different than that of Global North actors, thus underlining the muddled nature of the concept.

Mpox has been endemic in Central and West Africa for decades. At present, there are two clades: I and II. Clade I has more severe outcomes and has long been endemic in countries such as the DRC, and its subclade is responsible for the current resurgence. Clade II, however, is historically less severe. Its subclade was responsible for the 2022 outbreak that spread across countries outside the endemic zones. The 2022 outbreak instigated a shift in guidelines on improving surveillance and vaccinations, although mostly operationalised in nonendemic countries. Some high-income countries stockpiled vaccines, while at-risk populations in endemic zones were left without access.

As the disease spreads and calls to solidarity multiply, we are asking ourselves whether this current outbreak could have been avoided had solidarity been extended during the 2022 response, or indeed earlier, as the disease has been endemic for over 40 years. We find it useful to conceptualise solidarity in two ways, namely, *deep* solidarity and *responsive* solidarity.

Deep solidarity is rooted in the understanding that individual well-being is linked to collective well-being. The starting point for deep solidarity is not ‘I’ and what I may owe to others, but ‘we’ and how we can flourish. This relates to a deeper understanding of our being, one that is intrinsically inseparable and web-like. This understanding of deep solidarity places obligations on us, and in the context of global health, this could translate to equitable access to vaccines which not only serves the immediate populations but can also be a step towards employing protective mechanisms for the well-being of the whole. Another necessary step includes adopting deep preventative measures that address the driving factors behind outbreaks. These can be political, socioeconomic and/or structural challenges.

In the case of the DRC, there are challenges with basic health infrastructure including inadequate cold chains to house vaccines,^6^ which have slowed down the vaccination roll-out. For these populations, the viral spread has been fuelled by multiple factors, including inadequate water and sanitation services. In the epicentre, South Kivu, these factors have exacerbated the spread of mpox, particularly in children who are estimated to have not only been affected over twice as much as the wider population but are also experiencing worse outcomes associated with being immunocompromised due to malnutrition.^7^ This highlights both the need for deep solidarity, during and outside the times of disease outbreaks, to address the structural health challenges and not merely responsive solidarity which is activated to address crises or emergencies. Responsive solidarity is event-driven and reactionary, and can be emotionally charged and politically motivated. In the context of outbreaks in the Global South, responsive solidarity is often used as a utilitarian measure when the perceived risk is at the doorstep of populations in the Global North.

Responsive solidarity centres this notion of a common adversary that must be overcome, often marked by catchy familiar slogans like ‘we are all in this together’ or ‘no one is safe until we are all safe’. Without a deeper grounding, these phrases risk becoming rhetorical, and a disguised form of re-enacting or repeating the much-criticised ‘charity model’ of the 1980s that global health is trying to distance itself from. Current global health discourse and praxis exhibit an ambivalent relationship with solidarity as a normative ethical principle. Solidarity looks like an attractive concept to appeal to in times of emergencies and crises; however, there is a resistance, or daresay hostility, to undergirding global health justice and ethics on solidarity as was witnessed in the expunging of solidarity from the several iterations of the WHO-led pandemic agreement texts.

Solidarity, by definition, is an action concept. It is different from similar concepts like empathy or sympathy.^8 9^ It is, however, a late arrival in global health justice and ethics discourse; hence, the paucity of tools for enacting and holding actors accountable in their practice of solidarity. Interestingly, we can learn from communities in the Global South whose sociopolitical and ethical frameworks are deeply rooted in solidarity, for example, through the concepts of Igwebuike (Nigeria),^10^ Harambee (Kenya)^11^ and Ujamaa (Tanzania).^12^ These concepts come with binding obligations and duties. On the global level, there are conversations taking place on the institutionalisation of a legally binding form of solidarity as exemplified by the draft declaration on the right to international solidarity by the Office of the United Nations High Commissioner for Human Rights.

Deep solidarity inherently includes deep prevention, requiring engagement at the local, national, regional and international levels to strengthen prevention as part of preparedness. Achieving this would necessitate a fundamental reorganisation of relationships and systems that acknowledge the interconnectedness of health systems. This would not only promote equality and mutual well-being but would also bolster control and containment measures during PHEICs. Deep solidarity, therefore, involves providing populations in endemic regions with the same life-saving vaccines and treatments as populations in nonendemic zones, rather than the adoption of the ‘matter out of place’^13^ policies that we saw following the 2022 outbreak. This type of policy has meant focusing resources, including stockpiling vaccinations^14^ in spaces where mpox is deemed to be ‘out of place’, at the cost of implementing equitable and potentially lifesaving policies that forward and protect the whole.

The international response to mpox has been somewhat languid, failing to distribute vaccines to endemic zones in 2022, and again in the DRC in September 2023, allowing the outbreak to reach international crisis levels before practicing any sort of solidarity. This speaks to the limitations of responsive solidarity (ie, vaccine donation in outbreaks) when it does not spring from a deeply solidaristic foundation. Responsive solidarity, done well, is intertwined with, and stems from, deep solidarity. It is a balance between treating the symptoms and the disease. Vaccination is not a magic bullet.^15^ When deep solidarity is absent, responsive solidarity can be a dangerous façade that has grave implications for health outcomes. In its feigning and in rhetoric, it creates a false sense and false understanding of what solidarity means, as we see in the disalignment between the calls to solidarity and the response. It is, therefore, imperative that global health actors continue to conceptualise solidarity beyond rhetoric.

## Data Availability

There are no data in this work.
